# Loss of CIB2 Causes Profound Hearing Loss and Abolishes Mechanoelectrical Transduction in Mice

**DOI:** 10.3389/fnmol.2017.00401

**Published:** 2017-12-04

**Authors:** Yanfei Wang, Jie Li, Xuerui Yao, Wei Li, Haibo Du, Mingliang Tang, Wei Xiong, Renjie Chai, Zhigang Xu

**Affiliations:** ^1^Shandong Provincial Key Laboratory of Animal Cells and Developmental Biology, School of Life Sciences, Shandong University, Jinan, China; ^2^Shandong Provincial Collaborative Innovation Center of Cell Biology, Shandong Normal University, Jinan, China; ^3^School of Life Sciences, IDG/McGovern Institute for Brain Research, Tsinghua University, Beijing, China; ^4^Key Laboratory for Developmental Genes and Human Disease, Ministry of Education, Institute of Life Sciences, Southeast University, Nanjing, China; ^5^Co-Innovation Center of Neuroregeneration, Nantong University, Nantong, China; ^6^Jiangsu Province High-Tech Key Laboratory for Bio-Medical Research, Southeast University, Nanjing, China

**Keywords:** hearing loss, Usher syndrome, CIB2, knockout mice, stereocilia, mechanoelectrical transduction

## Abstract

Calcium and integrin-binding protein 2 (CIB2) belongs to a protein family with four known members, CIB1 through CIB4, which are characterized by multiple calcium-binding EF-hand domains. Among the family members, the *Cib1* and *Cib2* genes are expressed in mouse cochlear hair cells, and mutations in the human *CIB2* gene have been associated with nonsyndromic deafness DFNB48 and syndromic deafness USH1J. To further explore the function of CIB1 and CIB2 in hearing, we established *Cib1* and *Cib2* knockout mice using the clustered regularly interspaced short palindromic repeat (CRISPR)-associated Cas9 nuclease (CRISPR/Cas9) genome editing technique. We found that loss of CIB1 protein does not affect auditory function, whereas loss of CIB2 protein causes profound hearing loss in mice. Further investigation revealed that hair cell stereocilia development is affected in *Cib2* knockout mice. Noticeably, loss of CIB2 abolishes mechanoelectrical transduction (MET) currents in auditory hair cells. In conclusion, we show here that although both CIB1 and CIB2 are readily detected in the cochlea, only loss of CIB2 results in profound hearing loss, and that CIB2 is essential for auditory hair cell MET.

## Introduction

Usher syndrome (USH) is the most frequent form of inherited sensory deaf-blindness. It is characterized by hearing loss and vision defects with or without balance problems (Boughman et al., 1983). Based on the clinical symptoms, USH is classified into three types, namely USH1, USH2 and USH3. USH1 is the most severe type, which shows profound congenital deafness combined with retinitis pigmentosa and vestibular areflexia. USH2 is less severe and shows moderate congenital hearing impairment combined with retinitis pigmentosa and some balance problems. USH3, the least severe form, shows progressive hearing loss combined with retinitis pigmentosa and slight vestibular problems.

To date, 10 genes have been associated with USH, including *MYO7A* (Weil et al., 1995), *USH1C* (Bitner-Glindzicz et al., 2000; Verpy et al., 2000), *CDH23* (Bolz et al., 2001; Bork et al., 2001), *PCDH15* (Ahmed et al., 2001; Alagramam et al., 2001), *USH1G* (Weil et al., 2003), *CIB2* (Riazuddin et al., 2012), *USH2A* (Eudy et al., 1998), *ADGRV1* (Weston et al., 2004), *WHRN* (Ebermann et al., 2007) and *CLRN-1* (Joensuu et al., 2001). Furthermore, *PDZD7* was suggested to be a USH modifier and a contributor to digenic USH (Ebermann et al., 2010). Mutations in the USH genes have also been shown to cause nonsyndromic hearing loss. In the inner ear sensory hair cells, USH proteins bind to one another and form multiprotein complexes, and they are indispensable for the development, maintenance and function of hair cell stereocilia (Mathur and Yang, 2015). For example, CDH23 and PCDH15 interact with each other and form the so-called tip links, which connect the tips of shorter stereocilia to the sides of their taller neighboring stereocilia and play a pivotal role in hair cell mechanoelectrical transduction (MET; Kazmierczak et al., 2007).

Calcium and integrin-binding protein 2 (CIB2) is a newly discovered member of the USH protein family. Its biological function as well as its role in hearing remain largely unknown. Besides USH, mutations in the *CIB2* gene are also associated with nonsyndromic hearing loss DFNB48 (Riazuddin et al., 2012; Patel et al., 2015; Seco et al., 2016). CIB2 belongs to a family with four known members, CIB1 through CIB4, all of which contain multiple calcium-binding EF-hand domains (Gentry et al., 2005). As the prototype of this protein family, CIB1 has been intensively investigated, and it has been shown that CIB1 is involved in various cellular and physiological processes such as cell proliferation, apoptosis, cytoskeleton organization, angiogenesis and spermatogenesis (Leisner et al., 2005; White et al., 2006; Yuan et al., 2006; Naik et al., 2009; Heineke et al., 2010; Jarman et al., 2010; Zayed et al., 2010; Naik and Naik, 2011a,b; Kostyak et al., 2012). Mammalian CIB2 has been detected in skeletal muscle, the brain, the eye and the inner ear. In skeletal muscle, CIB2 colocalizes with the integrin 7B subunit at the sarcolemma as well as the neuromuscular junctions (NMJ) and the myotendinous junctions (MTJ; Häger et al., 2008). In the brain, CIB2 has mainly been detected in the hippocampus and cortex where it localizes together with the Golgi apparatus and dendrite markers (Blazejczyk et al., 2009). In the eye, CIB2 has been detected in the inner and outer segments of photoreceptor cells as well as retinal pigmented epithelium (RPE) cells (Riazuddin et al., 2012). In the inner ear, CIB2 has mainly been detected on hair cell stereocilia, where it binds to two other USH proteins—MYO7A and whirlin (Riazuddin et al., 2012).

The physiological function of CIB2 has been investigated using the zebrafish and fruit fly as models. In the zebrafish embryo, morpholinos against *Cib2* result in developmental defects such as microphthalmia, curled tail, hypopigmentation and edematous heart (Riazuddin et al., 2012). Hair cell patches in lateral-line neuromasts of morphants are markedly reduced, and FM1-43 dye uptake and microphonic potential measurements suggested that the MET components are abnormal (Riazuddin et al., 2012). In the fly, knockdown of *Cib2* expression through RNA interference (RNAi) causes significantly reduced photoresponse amplitude and impaired responses to visual stimuli (Riazuddin et al., 2012). Recently, during the preparation of our manuscript, two groups independently reported that *Cib2* knockout mice are profoundly deaf (Giese et al., 2017; Zou et al., 2017). It was also shown that CIB2 binds TMC1/2 and the MET currents are completely absent in *Cib2* knockout hair cells (Giese et al., 2017).

Among the four known *Cib* genes, the *Cib1* and *Cib2* genes are abundantly expressed in mouse cochlear hair cells. In the present work, *Cib1* and *Cib2* knockout mice were developed to examine their role in hearing transduction. Our data showed that *Cib2* disruption indeed causes hearing loss and abolishes the MET currents. We also found that the voltage-gated currents in *Cib2* knockout outer hair cells (OHCs) are slightly reduced. Furthermore, our results suggested that loss of CIB1 does not affect the auditory function in mice.

## Materials and Methods

### Generation of *Cib1* and *Cib2* Knockout Mice

Knockout mice were generated using the clustered regularly interspaced short palindromic repeat (CRISPR)-associated Cas9 nuclease (CRISPR/Cas9) genome editing technique as previously described (Yang et al., 2013). Briefly, C57BL/6 female mice (7–8 weeks old) were superovulated by intraperitoneally injecting pregnant mare serum gonadotropin (PMSG) and human chorionic gonadotrophin (hCG) and then mated to C57BL/6 male mice. The fertilized embryos (zygotes) were collected from the oviducts, and mixed *Cas9* mRNA (50 ng/μl) and small guide RNA (sgRNA; 25 ng/μl) were injected into the cytoplasm of zygotes with visible pronuclei in Chatot-Ziomek-Bavister (CZB) medium. The injected zygotes were then cultured in Quinn’s Advantage cleavage medium (*in vitro* Fertilization, Inc.) for 24 h, at which time 18–20 2-cell–stage embryos were transferred into the oviduct of a pseudopregnant ICR female mouse at 0.5 day post coitus (dpc). The accession numbers of the *Cib1* and *Cib2* cDNAs used to design sgRNA are NM_011870 and NM_019686, respectively. To determine the nucleotide sequence of mutated alleles, genomic DNA of F0 mice was amplified using the following primers: *Cib1* forward, 5′-GGA GGA TGA GGA GTT GGA-3′, *Cib1* reverse, 5′-CTG TGT GGG AAG GAG TGG-3′, *Cib2* forward, 5′-GGA TTG TGG AGG CTT TCT-3′ and *Cib2* reverse, 5′-ATG GCT CAC AAG ATG CTC-3′. DNA sequencing was then performed after TA cloning into plasmid pMD19T. In order to obtain F1 knockout mice, F0 mice were crossed with C57BL/6 mice and newborns were examined by Sanger sequencing.

### RNA Extraction and RT-PCR

Total RNA was extracted using TRIzol reagent (Invitrogen, Carlsbad, CA, USA) according to the manufacturer’s protocol, and 1 μg of total RNA was used as the template for reverse transcription (RT) using the PrimeScript RT reagent kit (Takara). Polymerase chain reaction (PCR) was then performed with the following primers: *Cib1* RT forward, 5′-ATG GGA GGT TCG GGC AGT CG-3′, *Cib1* RT reverse, 5′-TCA CAG GAC AAT CTT AAA GG-3′, *Cib2* RT forward, 5′-AAG AGA GGA TTG TGG AGG-3′, *Cib2* RT reverse, 5′-CAG ACT TGG TGA GTC GGG-3′, *Cib3* RT forward, 5′-ATG AGG CTG TTC TAT CGA TA-3′, *Cib3* RT reverse, 5′-GTC AGA TGC GGA TGT GGA AGG-3′, *Cib4* RT forward, 5′-ATG GGG CAG TGT TTA AGG TA-3′, and *Cib4* RT reverse, 5′-TCA GCA GCC CCA GAA GTG AA-3′, *β-actin* RT forward, 5′-ACG GCC AGG TCA TCA CTA TTG-3′, *β-actin* RT reverse, 5′-AGG GGC CGG ACT CAT CGT A-3′. The PCR products were separated by electrophoresis on agarose gels. Primers for quantitate-PCR are as follows: *Cib1* forward, 5′- AGC CTG AGC TTT GAG GAC TTC-3′, *Cib1* reverse, 5′- ACA TGC TGG AAC TCG GAA AGA-3′, *Cib2* forward, 5′- TTT CTC CGA GGA TGG CGA-3′, *Cib2* reverse, 5′- CAG ACT TGG TGA GTC GGG-3′, *Cib3* forward, 5′- GGA TGG TCA CAT GAC CTT AGA G-3′, *Cib3* reverse, 5′- GTC CCA TGC ACA GAT GTA GTT-3′, *Cib4* forward, 5′- TGG TTT CAT TGA TGA GGA GGA-3′ and *Cib4* reverse, 5′- AGA TCT GAC TCA CTC AGG ACA T-3′.

### Auditory Brainstem Response (ABR) Measurements

Mice were anesthetized intraperitoneally with 8.4 mg pentobarbital/100 g body weight. Body temperature was maintained at 37°C by placing the mice on an isothermal pad during testing and recovery from anesthesia. Electrodes were inserted subcutaneously at the vertex and pinna as well as near the tail. The stimulus generation, presentation, ABR acquisition and data management were coordinated using a RZ6 workstation and BioSig software (Tucker Davis Technologies, Inc.). Specific acoustic stimuli (clicks or tone bursts) were generated using high-frequency transducers. At each sound level, 512 responses were sampled and averaged. ABR thresholds were obtained for each animal by reducing the stimulus intensity in 10 dB SPL steps to identify the lowest intensity at which all ABR waves were detectable.

### Distortion Product Otoacoustic Emission (DPOAE) Measurement

Mice were anesthetized and maintained as mentioned above. Two sine wave tones of different frequencies (F2 = 1.2 × F1) were presented for 1 s durations ranging from 20 dB to 80 dB SPL in 10 dB steps. The emitted acoustic signal was picked up by the microphone and digitized, and the magnitude of the distortion product (2 × F1 − F2) was determined. The surrounding noise floor was calculated by averaging adjacent frequency bins around the distortion product frequency. Distortion product otoacoustic emission (DPOAE) thresholds were calculated when the signal was at least 5 dB SPL above the noise floor.

### Whole-Mount Immunostaining

All steps were performed at room temperature unless otherwise indicated. Mouse basilar membrane was dissected and fixed with cold 4% paraformaldehyde (PFA) in PBS for 30 min, then permeabilized and blocked with PBT1 (0.1% Triton X-100, 1% BSA and 5% heat-inactivated goat serum in PBS, pH 7.3) for 30 min. Samples were incubated overnight at 4°C with mouse anti-CIB2 polyclonal antibody (Abnova, Cat. No. H00010518-A01, 1:100 diluted) in PBT1 followed by incubation with Alexa Fluor^®^ 488 donkey anti-mouse secondary antibody (Invitrogen, Cat. No. A21202, 1:300 diluted) in PBT2 (0.1% Triton X-100 and 0.1% BSA in PBS) for 1 h. After that, samples were incubated with TRITC-conjugated phalloidin (Sigma-Aldrich, Cat. No. P1951) in PBS for 30 min, then mounted in PBS/glycerol (1:1) and imaged with a confocal microscope (LSM 700, Zeiss, Germany) and a 40-fold objective. Immunostaining was performed at least three times for each genotype and age of mice tested.

### Scanning Electron Microscopy (SEM)

Mouse inner ears were dissected and fixed with 2.5% glutaraldehyde in 0.1 M phosphate buffer overnight at 4°C. Cochleae were dissected out of the temporal bone, post-fixed with 1% osmium tetroxide in 0.1 M phosphate buffer at 4°C for 2 h, and dehydrated in ethanol and critically point dried using a Leica EM CPD300 (Leica, Germany). Samples were then mounted and sputter coated with platinum (15 nm) using a Cressington 108 sputter coater (Cressington, United Kingdom) and imaged using a Quanta250 field-emission scanning electron microscope (FEI, Netherlands).

### Electrophysiology

The apical-middle part of cochleae was acutely isolated and immobilized under a cross of dental floss. Whole cell recordings were carried out with an electrophysiology amplifier (HEKA, EPC-10 USB) running the PatchMaster software. Borosilicate glass filaments (Sutter) were made with a pipette puller (Sutter, P-2000) and polished with an MF-830 microforge (Narishige) to resistances of 3–5 MOhm. The recording solution contained 144 mM NaCl, 0.7 mM NaH_2_PO4, 5.8 mM KCl, 1.3 mM CaCl_2_, 0.9 mM MgCl_2_, 5.6 mM glucose and 10 mM H-HEPES (pH 7.4). The pipette solution contained 140 mM KCl, 1 mM MgCl_2_, 0.1 mM EGTA, 2 mM MgATP, 0.3 mM Na_2_GTP and 10 mM H-HEPES, pH7.2. The MET currents were evoked using a fluid jet from a pipette (tip diameter of 5–10 μm). The pipette tip delivering the fluid jet was positioned by facing the staircase side of the hair bundle at a distance of around 5 μm to elicit saturating MET currents. The 40 Hz sinusoidal force stimuli were generated by a 27-mm-diameter piezoelectric disc controlled by the HEKA amplifier and driven by a homemade piezo amplifier.

### Endocochlear Potential (EP) Recording

For stable endocochlear potential (EP) recording, 1-month-old mice were used. The mice were anesthetized by injection intraperitoneally with 4% pentobarbital sodium in saline. The left cochlea was exposed at its ventrolateral side, then the bulla near the round window was carefully removed to expose the round window. The sharp glass electrodes were pulled by pipette puller (Sutter, P-2000) with a tip-diameter of 0.1–0.2 μm. The electrode filled with 150 mM KCl was insert into the scala media through the round window controlled by a micromanipulator (Luigs&Neumann, mini 25). An Ag-AgCl reference electrode was placed into the neck muscle. The baseline of potential was offset to zero when the pipette tip reached to the round window before penetration. The data were collected by a patch-clamp amplifier (Axon, 700B) and an I/O interface (ITC18) that were powered with an electrophysiology software (WinWCP, University of Strathclyde Glasgow).

### Statistical Analysis

Data are shown as means ± standard deviations. Student’s *t*-test was used for statistical analysis, and *p* < 0.05 was considered statistically significant.

## Results

### Generation of *Cib1* and *Cib2* Knockout Mice

**Figure 1:**
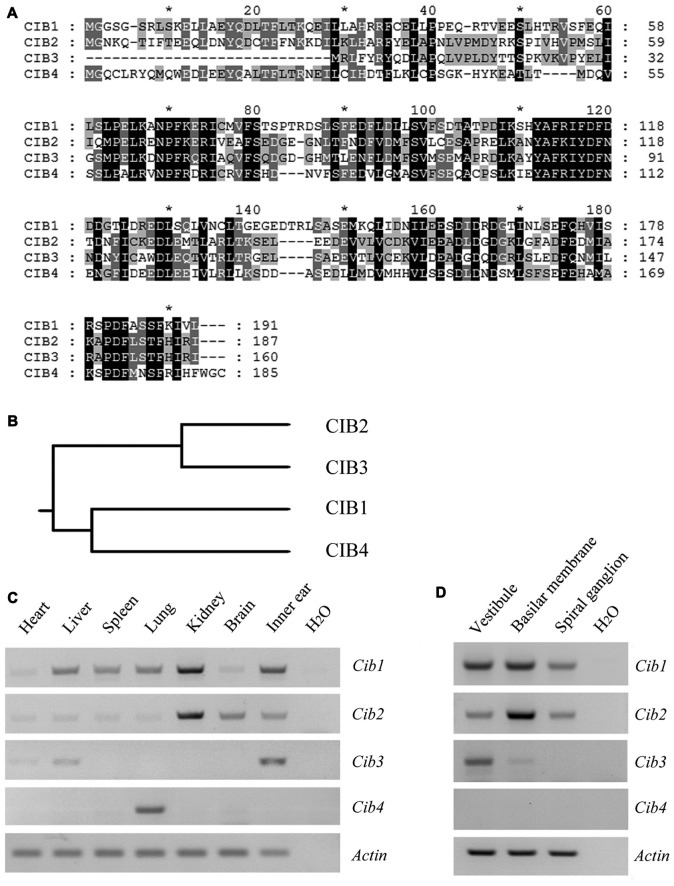
Expression of *Cib1*, *Cib2*, *Cib3* and *Cib4* in different mouse tissues. **(A)** Amino acid sequences of *Mus musculus* calcium and integrin-binding protein 1 (CIB1), CIB2, CIB3 and CIB4 were aligned using the CLUSTALW multiple sequence alignment program. **(B)** The phylogenic tree of *Mus musculus* CIB1, CIB2, CIB3 and CIB4 was constructed using the rooted phylogenetic tree with branch length (UPGMA) method. **(C)** Expression of *Cib1*, *Cib2*, *Cib3* and *Cib4* in different tissues of 6-month-old mice was determined by RT-PCR. **(D)** Expression of *Cib1*, *Cib2*, *Cib3* and *Cib4* in the vestibule, basilar membrane and spiral ganglion cells of P2 mice was determined by RT-PCR. *β-actin* was included as the internal control.

CIB2 belongs to a family with four known members, CIB1 through CIB4, which show significant similarity to each other (Figure 1A). In this protein family, CIB2 is more closely related to CIB3 (Figure 1B). RT-PCR results showed that *Cib1* and *Cib2* are ubiquitously expressed in various mouse tissues, whereas *Cib3* and *Cib4* have more restricted expression patterns. All *Cib* genes except *Cib4* were detected in the inner ear (Figure 1C). Further examination revealed that in the inner ear, *Cib1* and *Cib2* are expressed in the vestibule, basilar membrane and spiral ganglion, whereas *Cib3* is mainly detected in the vestibule (Figure 1D). The similar expression pattern of *Cib1* and *Cib2* in the inner ear suggests that both genes might play important roles in hearing, hence we focused on these two genes in the following work.

**Figure 2:**
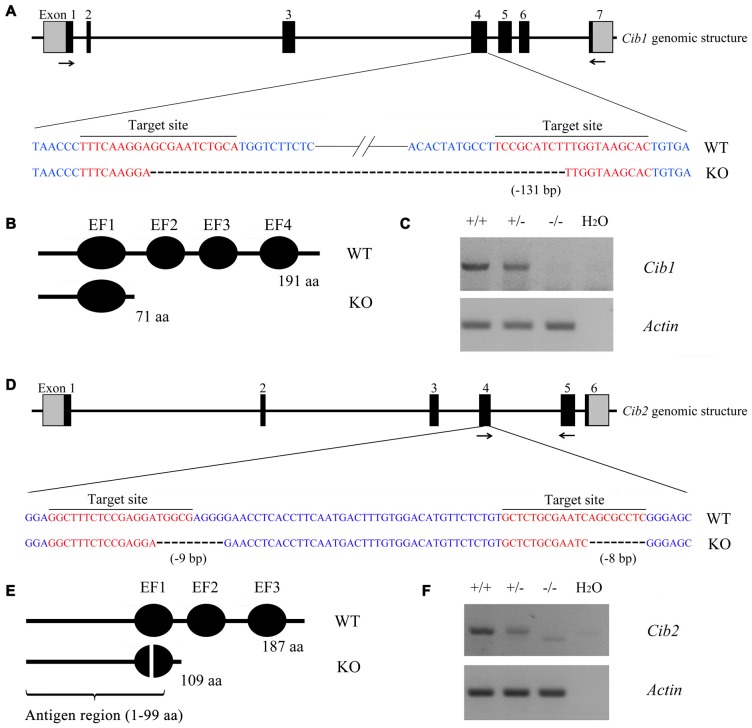
Construction of *Cib1* and *Cib2* knockout mice.** (A)** The schematic drawing of the strategy for *Cib1* gene disruption. The target sites of clustered regularly interspaced short palindromic repeat (CRISPR)-Cas9 small guide RNAs (sgRNAs) in the *Cib1* gene are indicated in red, and the deleted region in the *Cib1* gene of knockout mice is indicated by dashes. The positions of RT-PCR primers are indicated by arrows. **(B)** The schematic drawing of the domain structure of CIB1 in wildtype and knockout mice. **(C)** The expression of *Cib1* mRNA in the inner ear of P60 mice was determined by RT-PCR. *β-actin* was included as the internal control. **(D)** The schematic drawing of the strategy for *Cib2* gene disruption. The target sites of CRISPR-Cas9 sgRNAs in the *Cib2* gene are indicated in red, and the deleted regions in the *Cib2* gene of knockout mice are indicated by dashes. The positions of RT-PCR primers are indicated by arrows. **(E)** The schematic drawing of the domain structure of CIB2 in wildtype and knockout mice. **(F)** The expression of *Cib2* mRNA in the inner ear of P60 mice was determined by RT-PCR. *β-actin* was included as the internal control.

In order to investigate the functions of CIB1 and CIB2 in hearing, we developed knockout mice using the CRISPR/Cas9 genome editing technique. The mouse *Cib1* gene contains seven exons encoding 191 amino acids, and two sgRNAs were designed to target exon 4 (Figure 2A). DNA sequencing revealed that a deletion of 131 bp was introduced in exon 4 in *Cib1* knockout mice, which causes a premature translational stop and gives rise to a potentially truncated CIB1 protein of 71 amino acids (Figure 2B). The mouse *Cib2* gene contains six exons encoding 187 amino acids, and two sgRNAs were designed to target exon 4 (Figure 2D). DNA sequencing revealed that two deletions (9 bp and 8 bp, respectively) were introduced into exon 4 in *Cib2* knockout mice, and these cause a premature translational stop that gives rise to a potentially truncated protein of 109 amino acids (Figure 2E). Three *Cib2* transcript variants have been reported that result from alternative splicing (Riazuddin et al., 2012). Given that exon 4 is a common exon for all three transcript variants, the deletions will affect all of the three known CIB2 isoforms. RT-PCR was then performed to confirm the disruption of *Cib1* and *Cib2* mRNA in knockout mice. A single PCR fragment was amplified in the wild type and heterozygous mice, whereas this fragment was not detected in the homozygous knockout mice (Figures 2C,F). A smaller fragment corresponding to the deletion was detected in *Cib2* homozygous knockout mice at very low level (Figure 2F), whereas no such fragment was detected in *Cib1* homozygous knockout mice (Figure 2C), possibly caused by nonsense-mediated mRNA decay (NMD). We then utilized a specific anti-CIB2 antibody to examine the expression of CIB2 protein in *Cib2* knockout mice. This antibody recognizes the first 99 amino acids of CIB2, hence could detect both the wildtype as well as the potentially truncated CIB2 (Figure 2E). Although the antibody does not work for western blot, immunostaining result suggests that no truncated CIB2 protein is expressed in the *Cib2* knockout mice (see below). Taken together, our results indicate that *Cib1* and *Cib2* expression is successfully disrupted in the *Cib1* and *Cib2* knockout mice, respectively.

### Loss of CIB2 but Not CIB1 Causes Profound Hearing Loss in Mice

**Figure 3:**
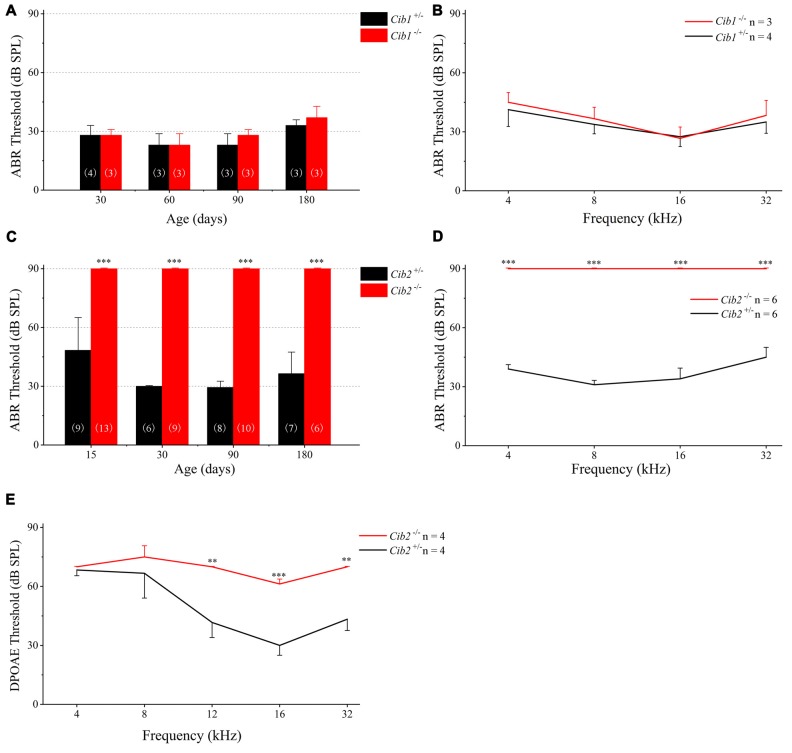
Hearing threshold is elevated in *Cib2* knockout mice, but not in *Cib1* knockout mice.** (A)** The auditory brainstem response (ABR) thresholds for click stimuli in *Cib1*^+/−^ and *Cib1*^−/−^ mice at different ages. **(B)** The ABR thresholds for pure tone stimuli in 1-month-old *Cib1*^+/−^ and *Cib1*^−/−^ mice. **(C)** The ABR thresholds for click stimuli in *Cib2*^+/−^ and *Cib2*^−/−^ mice at different ages. **(D)** The ABR thresholds for pure tone stimuli in 1-month-old *Cib2*^+/−^ and *Cib2*^−/−^ mice. **(E)** The distortion product otoacoustic emission (DPOAE) thresholds for pure tone stimuli in 1-month-old *Cib2*^+/−^ and *Cib2*^−/−^ mice. The numbers of animals for each group used in the experiments are indicated. The differences were evaluated by Student’s *t*-test (**p < 0.01; ***p < 0.001).

We evaluated the auditory function of *Cib1* and *Cib2* knockout mice by performing ABR measurements. The homozygous *Cib1* knockout mice had normal hearing thresholds in response to click stimuli at all ages examined up to 6 months (Figure 3A). Similar results were obtained when pure tone stimuli of different frequencies were used, suggesting that CIB1 is not necessary for auditory function in mice (Figure 3B). We then examined the expression of *Cib* members in the inner ear of* Cib1* knockout mice by performing quantitate PCR. The result showed that *Cib3* is upregulated in the inner ear of *Cib1* knockout mice, suggesting that CIB3 might compensate for the loss of CIB1 (Supplementary Figure S1).

In contrast to *Cib1*, homozygous *Cib2* knockout mice showed significant hearing threshold elevation to click stimuli when examined as early as postnatal day 14 (P14; Figure 3C). In fact, the loudest sound stimuli (90 dB) used in our experiment could not evoke any response in homozygous *Cib2* knockout mice. Significant threshold elevation was also observed when pure-tone sound stimuli of different frequencies were used (Figure 3D). DPOAE measurements were then performed to examine the function of OHCs in *Cib2* knockout mice. The DPOAE threshold of homozygous *Cib2* knockout mice was significantly elevated compared to heterozygous mice, suggesting that there are OHC function deficits in *Cib2* knockout mice (Figure 3E). Taken together, our data suggest that hearing function is severely affected by loss of CIB2 but not by loss of CIB1. Hence we focused on CIB2 in the following work.

### Loss of CIB2 Affects Stereocilia Development in Mice

**Figure 4:**
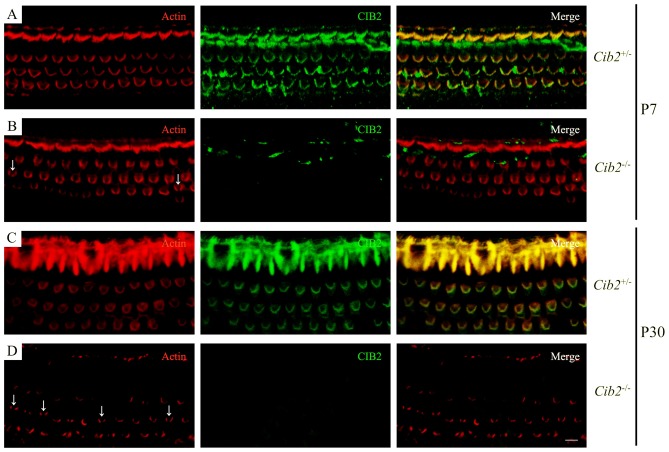
Whole-mount immunostaining in *Cib2* knockout mice. Shown are single confocal sections. CIB2 immunoreactivity was visualized with FITC-conjugated secondary antibody, and F-actin was visualized with rhodamine-conjugated phalloidin. **(A)** P7 *Cib2*^+/−^ mice. **(B)** P7 *Cib2*^−/−^ mice. **(C)** P30 *Cib2*^+/−^ mice. **(D)** P30 *Cib2*^−/−^ mice. The images were taken at the middle turn of the cochlea. Stereocilia bundle fragmentation was indicated by arrows. Scale bars: 5 μm.

We examined stereocilia morphology by performing whole-mount staining. The OHC stereociliary bundles of homozygous *Cib2* knockout mice are disorganized at P7, the earliest age examined, whereas inner hair cell (IHC) stereociliary bundles are largely unaffected. In some *Cib2*^−/−^ OHCs, stereociliary bundle fragmentation was observed (Figures 4A,B). At P30, the disorganization of *Cib2*^−/−^ OHC stereociliary bundles is further exacerbated (Figures 4C,D). Consistent with previous report (Riazuddin et al., 2012), CIB2 immunoreactivity is detected in the stereociliary bundles of heterozygous mice but is undetectable in *Cib2* homozygous knockout mice (Figures 4A–D).

**Figure 5:**
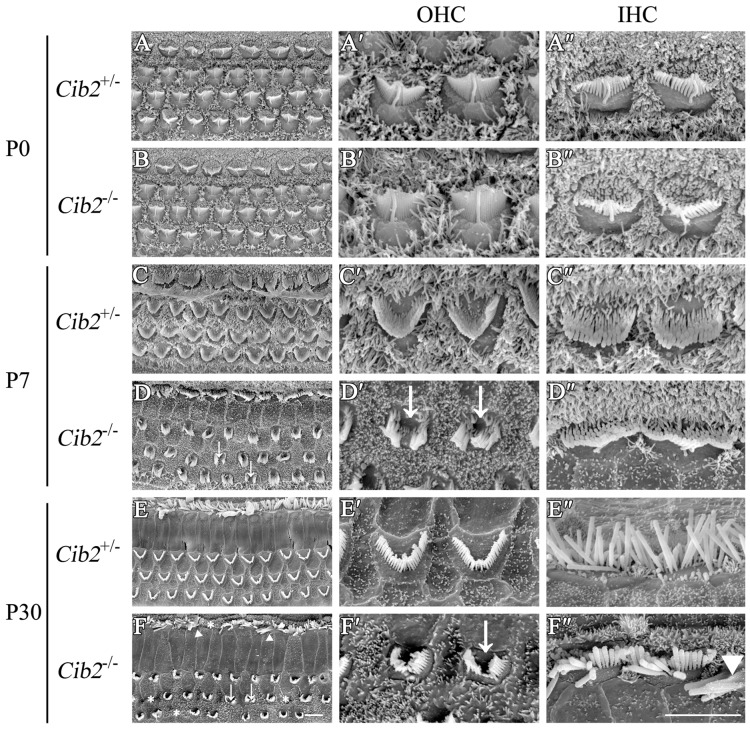
Stereocilia are disorganized in *Cib2* knockout mice. **(A–F)** Low-magnification scanning electron microscopy (SEM) images of cochlear hair bundles from mice of different genotypes and ages as indicated. **(A′–F′)** High-magnification SEM images of outer hair cell (OHC) hair bundles from mice of different genotypes and ages. **(A″–F″)** High-magnification SEM images of inner hair cell (IHC) hair bundles from mice of different genotypes and ages. The images were taken at the apical-middle turn of the cochlea. Stereociliary bundle fragmentation is indicated by arrows, stereocilia loss is indicated by asterisks, and stereocilia fusion is indicated by triangles. Scale bars, 5 μm.

The effect of *Cib2* disruption on stereocilia development was further examined by scanning electron microscopy (SEM). At P0, the stereocilia morphology of *Cib2*^−/−^ mice is indistinguishable from that of *Cib2*^+/−^ mice (Figures 5A–B″). However, at P7 *Cib2*^−/−^ OHC stereocilia are largely disorganized, and fragmentation was observed in some *Cib2*^−/−^ OHC stereocilia bundles (Figures 5C–D″). At P30, the disorganization of *Cib2*^−/−^ OHC stereocilia is increased, and some *Cib2*^−/−^ OHC completely lose their stereocilia (Figures 5E–F″). Additionally, the IHCs of *Cib2*^−/−^ mice at this age also show morphological abnormalities, with stereocilia fusion and occasional stereocilia loss (Figures 5E–F″).

**Figure 6:**
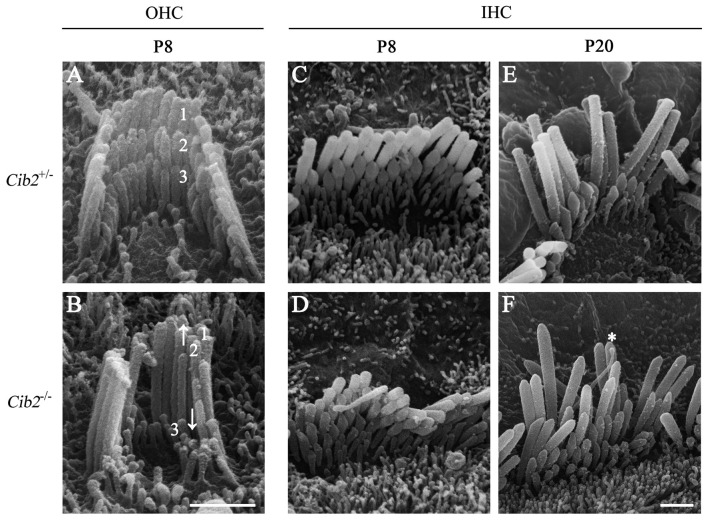
Hair bundle development is affected in *Cib2* knockout mice. **(A–F)** High-magnification SEM images of cochlear hair bundles from mice of different genotypes and ages as indicated. The images were taken at the apical-middle turn of the cochlea. Three rows of stereocilia are indicated by numbers. Over-grown and retracted stereocilia are indicated by arrows up and down, respectively. The kinocilium that does not regress properly is indicated by asterisk. Scale bars, 1 μm.

Further SEM examination at higher magnification showed that at P8 the second row stereocilia of *Cib2*^−/–^ OHCs are over-grown to the height close to the first row, whereas the third row are largely retracted, resulting in the loss of staircase architecture (Figures 6A,B). In contrast, the staircase architecture of *Cib2*^−/−^ IHC stereocilia maintains despite that the second and third row stereocilia are over-grown, which is more obvious at P20 (Figures 6C–F). Furthermore, the kinocilium is not retracted properly in *Cib2*^−/−^ IHC (Figure 6F). Taken together, our data suggest that loss of CIB2 affects stereocilia development in mice.

### Loss of CIB2 Abolishes MET Currents

**Figure 7:**
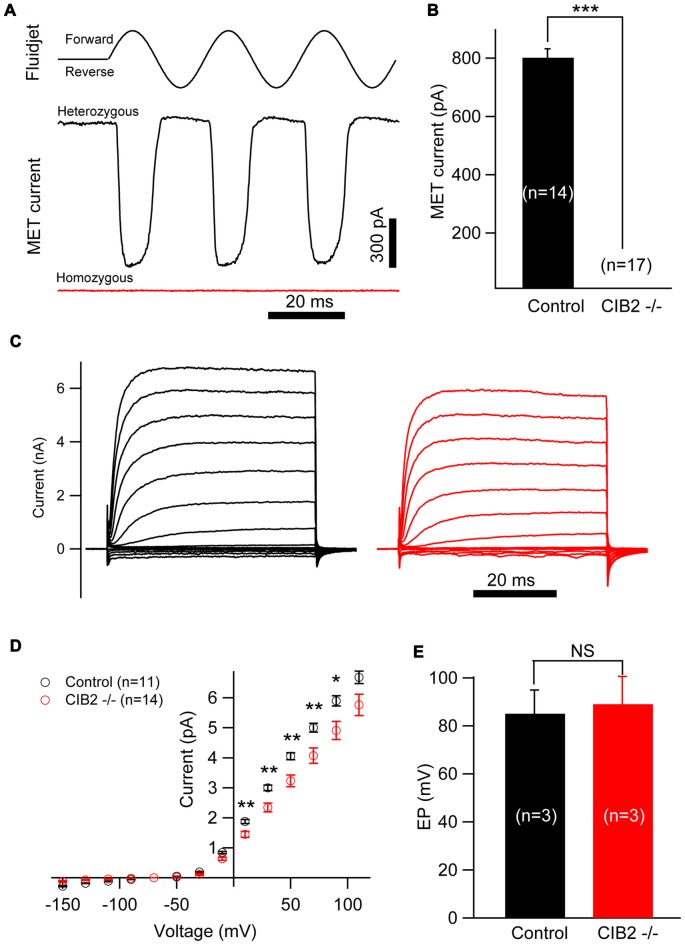
Mechanosensitivity of hair cells is lost in *Cib2* knockout mice. **(A)** Mechanoelectrical transduction (MET) current was examined in OHCs from control and *Cib2* knockout mice. A fluid jet system that drives a sinusoidal deflection of hair bundles was used to evaluate the saturating MET current from hair cells. **(B)** The average peak current was 829.2 pA in control OHCs but absent in knockout OHCs. **(C)** Voltage-gated current was recorded from control and *Cib2* knockout OHCs. The membrane potential was changed from −150 mV to +110 mV in 20 mV steps. **(D)** Current–voltage (I-V) curves were drawn from data similar to **(C)**, indicating reduced membrane potassium currents in *Cib2* knockout OHCs. **(E)** The average endocochlear potential (EP) was around 85 mV in control and 89 mV in *Cib2* knockout mice. In all panels, data were collected from three control heterozygous mice (shown in black) and three *Cib2* knockout mice (shown in red). The age of mice is P7 **(A–D)** or P30 **(E)**. The numbers of cells used are shown in each panel. The differences were evaluated by Student’s *t*-test (*p < 0.05; **p < 0.01; ***p < 0.001).

As CIB2 is largely detected in the hair bundles and contributes to stereocilia development, we asked whether CIB2 plays a role in MET in hair cells. By applying fluid jet stimulation that generally deflects hair bundles in a physiological manner, we recorded the saturating MET currents of OHCs. Strikingly, the MET current is completely abolished in P7 *Cib2* knockout OHCs. In contrast, there is a large MET current around 800 pA recorded in control OHCs (Figures 7A,B). Lack of MET current in *Cib2* knockout OHCs is not caused by stereocilia disorganization, since we always chose OHCs with relatively normal hair bundle morphology for recordings. Furthermore, we measured MET currents in P1 and P4 *Cib2* knockout OHCs whose stereocilia are less affected by *Cib2* disruption, and obtained similar results (Supplementary Figures S2A,B). *Cib2* knockout IHCs have more intact stereocilia compared to OHCs, and we found that MET currents are also absent in P7 *Cib2* knockout IHCs (data not shown). Taken together, our data suggest that CIB2 disruption abolishes MET currents in auditory hair cells. In contrast, reverse-polarity currents could be recorded in P4 *Cib2* knockout OHCs (Supplementary Figure S3), suggesting that CIB2 is not necessary for Piezo2-dependent reverse-polarity currents (Wu et al., 2017).

Interestingly, we found that the voltage-gated currents in P7 *Cib2* knockout OHCs are slightly reduced when compared to the OHCs collected from the littermate controls in the same cage (Figures 7C,D). The reduction of voltage-gated currents was not observed in P1 and P4 *Cib2* knockout OHCs (Supplementary Figure S4). Accordingly, we also examined the EP of *Cib2* knockout mice, which indicated that *Cib2* knockout mice maintain a normal potential in the scala media (Figure 7E).

## Discussion

*CIB2* is an important deafness gene whose mutations are associated with nonsyndromic deafness DFNB48 and syndromic deafness USH1J (Riazuddin et al., 2012; Patel et al., 2015; Seco et al., 2016), but its biological function and the mechanism through which *CIB2* mutations cause hearing loss remain elusive. Here we show that loss of CIB2 in mice affects MET as well as stereocilia development and results in profound hearing loss.

CIB2 belongs to a protein family with four known members, CIB1 through CIB4, that are characterized by multiple calcium-binding EF-hand domains (Gentry et al., 2005). RT-PCR experiments revealed that *Cib1* and *Cib2* are expressed in the vestibule, basilar membrane and spiral ganglion, whereas *Cib3* is mainly expressed in the vestibule. The fourth member *Cib4* was not detected in the inner ear. In all the mouse tissues examined, *Cib4* expression was only detected in the lung. The absence of *Cib4* expression in the mouse inner ear contradicts the recent report that *Cib4* is expressed in mouse cochlea and vestibule (Giese et al., 2017). We used two different pairs of primers to examine *Cib4* expression, both of which could not detect the expression of *Cib4* in mouse inner ear. Consistent with our finding, the RNA transcriptome sequencing result revealed extremely low level of *Cib4* expression in mouse inner ear (SHIELD^1^; Shen et al., 2015). It is worth noticing that in the present work we examined the expression of CIBs only at the mRNA level, which might not faithfully represent the protein expression level. Nevertheless, our data suggest that *Cib1*, *Cib2* and *Cib3* are expressed abundantly in mouse inner ear, whereas* Cib4* is expressed at very low level, if at all, in the inner ear.

The expression of *Cib1* and *Cib2* in the cochlea is consistent with the RNA transcriptome sequencing result, which also suggests that the expression level of *Cib2* in cochlear hair cells is about 10-fold greater than that of *Cib1* (SHIELD^1^; Shen et al., 2015). The relatively low expression level of *Cib1* in the cochlear hair cells might explain the fact that disruption of the *Cib1* gene does not affect auditory function in mice, since loss of CIB1 might be compensated for by other CIB members. Indeed, quantitate PCR result showed that *Cib3* expression is upregulated in the inner ear of *Cib1* knockout mice, suggesting that CIB3 might compensate for loss of CIB1. Similar scenario has been reported in platelets of *Cib1* knockout mice with *Cib1* exon 4 and 5 deleted through homologous recombination (Yuan et al., 2006; Denofrio et al., 2008).

In contrast to *Cib1*, *Cib2* disruption results in profound hearing loss in mice, suggesting an indispensable role of CIB2 in hearing. Similar phenotype was recently reported by two other groups using different *Cib2* mutant mice (Giese et al., 2017; Zou et al., 2017). *Cib2*^*tm1a*^ mice contain a gene trap cassette between *Cib2* exon 3 and 4 that consists of *lacZ* and neomycin resistance genes (Giese et al., 2017; Zou et al., 2017). Crossing *Cib2^*tm1a*^* mice with Cre-expressing mice results in the deletion of the neomycin cassette and exon 4, giving rise to *Cib2^*tm1b*^* mice (Giese et al., 2017). A deafness-associated missense mutation, p.F91S, was introduced into mice to give rise to *Cib2^*F91S*^* knockin mice (Giese et al., 2017). Similar to our *Cib2* knockout mice, all these *Cib2* mutant mice show profound hearing loss, strongly suggesting that CIB2 plays an important role in hearing.

CIB2 contains three EF-hand domains, and the last two EF-hands could bind calcium (Blazejczyk et al., 2009). It has been shown that calcium plays important roles in hair cell MET (Mammano et al., 2007; Ceriani and Mammano, 2012). Calcium enters hair cells through MET channels and is necessary for the adaptation of the channels. CIB2 has been shown to inhibit ATP-induced calcium release in transiently transfected COS-7 cells, which might result from its calcium-buffering ability (Riazuddin et al., 2012). Moreover, CIB2 might regulate calcium release by interacting with IP_3_ receptors, just as its paralog CIB1 does (White et al., 2006; Hennigs et al., 2008). Loss of CIB2 might therefore result in dysregulation of intracellular calcium in hair cells, which in turn would affect MET. Interestingly, our data showed that MET currents are completely abolished in mice lacking functional CIB2, which was also observed in mice lacking potential MET components such as TMC1/TMC2 and TMIE (Kawashima et al., 2011; Kim et al., 2013; Pan et al., 2013; Zhao et al., 2014). This suggests that CIB2 might play important roles in MET in addition to intracellular calcium regulation. In line with this, CIB2 immunoreactivity localizes near the tips of shorter stereocilia (Riazuddin et al., 2012; Giese et al., 2017), a place where MET channels reside (Beurg et al., 2009). Giese et al. (2017) reported that CIB2 could interact with TMC1 and TMC2, and MET currents are absent in *Cib2^*tm1a*^*/*Cib2^*tm1b*^* and *Cib2^*F91S*^* mice, raising the possibility that CIB2 might be directly involved in MET activity. The association between CIB2 and MET warrants further investigation.

Interestingly, the voltage-gated current is slightly reduced in the OHCs of *Cib2* knockout mice, whereas the EP was unaffected. Several voltage-regulated ion channels have been shown to play important roles in hair cells, including the voltage-gated K^+^ channel KCNQ4 and the Ca^2+^-activated K^+^ channel (BK channel; Ruttiger et al., 2004; Kharkovets et al., 2006). Mutations or disruptions in genes coding for KCNQ4 or BK channel cause hearing loss in both humans and mice (Coucke et al., 1999; Kubisch et al., 1999; Ruttiger et al., 2004; Kharkovets et al., 2006). Given the fact that CIB2 has Ca^2+^ buffering ability, loss of CIB2 might increase intracellular calcium concentration. On the other hand, CIB2 disruption abolishes MET currents, hence decreases calcium influx into hair cells. Although the net effect is unclear, loss of CIB2 might change intracellular calcium concentration, thus affect voltage-gated current through Ca^2+^- or voltage-gated channels. Furthermore, our data show that the voltage-gated current is reduced in P7* Cib2* knockout OHCs, but not in P1 or P4* Cib2* knockout OHCs, suggesting that CIB2 modulation on voltage-gated current is developmental stage-dependent. The significance of the role of CIB2 in this process remains elusive.

We also found that stereocilia development is affected in *Cib2* knockout mice. Stereocilia bundle fragmentation is seen in many *Cib2* knockout OHCs, and stereocilia fusion could be observed in some *Cib2* knockout IHCs. Strikingly, over-growth of shorter row stereocilia is observed in *Cib2* knockout OHCs, and similar scenario happens in *Cib2* knockout IHCs to a lesser extent. This results in the loss of staircase architecture in *Cib2* knockout OHC stereocilia bundle, but not IHC stereocilia bundle. Intracellular calcium has been shown to affect actin cytoskeleton dynamics in many cell types such as neutrophils and neurons (Downey et al., 1990; Welnhofer et al., 1999; Hutchins et al., 2013), and it was recently suggested that calcium influx through the MET channels controls the remodeling of shorter stereocilia (Vélez-Ortega et al., 2017). Hence, the potential changes of intracellular Ca^2+^ caused by loss of CIB2 is possibly responsible for the defects in stereocilia development seen in the knockout mice. Alternatively, CIB2 might regulate stereocilia development through interaction with other stereociliary proteins, such as whirlin and MYO7A (Riazuddin et al., 2012). Mutations in the genes encoding whirlin and MYO7A have been shown to affect stereocilia development, although the stereocilia deficits in these mutant mice are different from those of *Cib2* knockout mice (Self et al., 1998; Holme et al., 2002). At present it remains unclear why loss of CIB2 affects OHC and IHC stereocilia differently, and why it affects the second row and third row OHC stereocilia differently, too. Further investigation is needed to clarify the mechanism by which CIB2 regulates stereocilia development.

## Ethics Statement

All animal experiments were approved by the Ethics Committee of Shandong University (Permit Number: LL-201502028) and conducted accordingly.

## Author Contributions

RC and ZX conceived the project and designed the experiments. YW, JL, XY, WL and HD performed the experiments. YW, JL, XY, MT and WX analyzed the data. WX, RC and ZX wrote the manuscript.

## Conflict of Interest Statement

The authors declare that the research was conducted in the absence of any commercial or financial relationships that could be construed as a potential conflict of interest.

## Footnotes

^1^https://shield.hms.harvard.edu
