# Supplementary material for: Loss of CIB2 Causes Profound Hearing Loss and Abolishes Mechanoelectrical Transduction in Mice
Source: Front Mol Neurosci. 2017 Dec 4;10:401. doi: 10.3389/fnmol.2017.00401 (PMC5722843; doi:10.3389/fnmol.2017.00401)
Supplement: Supplementary file 1 [file Data_Sheet_1.pdf]

Loss of CIB2 causes profound hearing loss and abolishes mechanoelectrical transduction in mice

Yanfei Wang[1,2†], Jie Li[3†], Xuerui Yao[1,2†], Wei Li[1,2], Haibo Du[1,2], Mingliang Tang[4,5], Wei Xiong[3], Renjie Chai[4,5,6*], Zhigang Xu[1,2*]

[1]Shandong Provincial Key Laboratory of Animal Cells and Developmental Biology, Shandong University School of Life Sciences, Jinan, China.

[2]Shandong Provincial Collaborative Innovation Center of Cell Biology, Shandong Normal University, Jinan, China

[3]School of Life Sciences, IDG/McGovern Institute for Brain Research, Tsinghua University, Beijing, China

[4]Key Laboratory for Developmental Genes and Human Disease, Ministry of Education, Institute of Life Sciences, Southeast University, Nanjing, China

[5]Co-Innovation Center of Neuroregeneration, Nantong University, Nantong, China

[6]Jiangsu Province High-Tech Key Laboratory for Bio-Medical Research, Southeast University, Nanjing, China

[†]Contributed equally to this work

[*]Corresponding author:

Renjie Chai, E-mail: renjiec@seu.edu.cn, Tel/Fax: 86-25-83790971

Zhigang Xu, E-mail: xuzg@sdu.edu.cn, Tel/Fax: 86-531-88362647

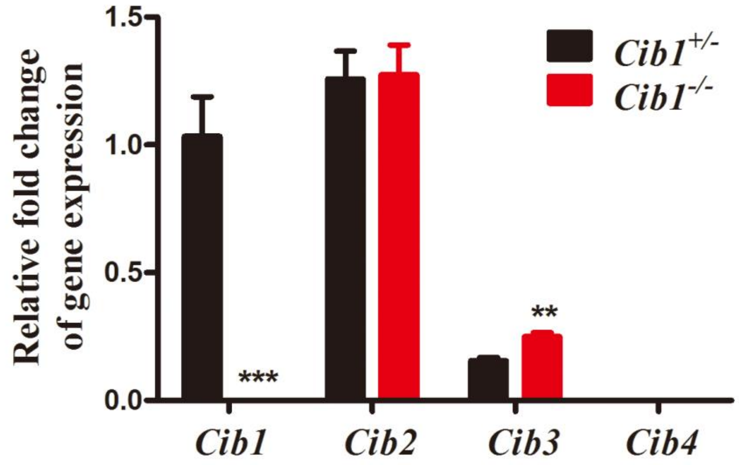

Figure S1. Expression of *Cib1*, *Cib2*, *Cib3*, and *Cib4* in the inner ear of *Cib1* knockout mice. Total RNA of P30 mouse inner ear was extracted and reverse transcribed, and expression of *Cib* genes were examined with quantitate PCR. The differences were evaluated by Student's t-test ($^{**}p < 0.01$; $^{***}p < 0.001$).

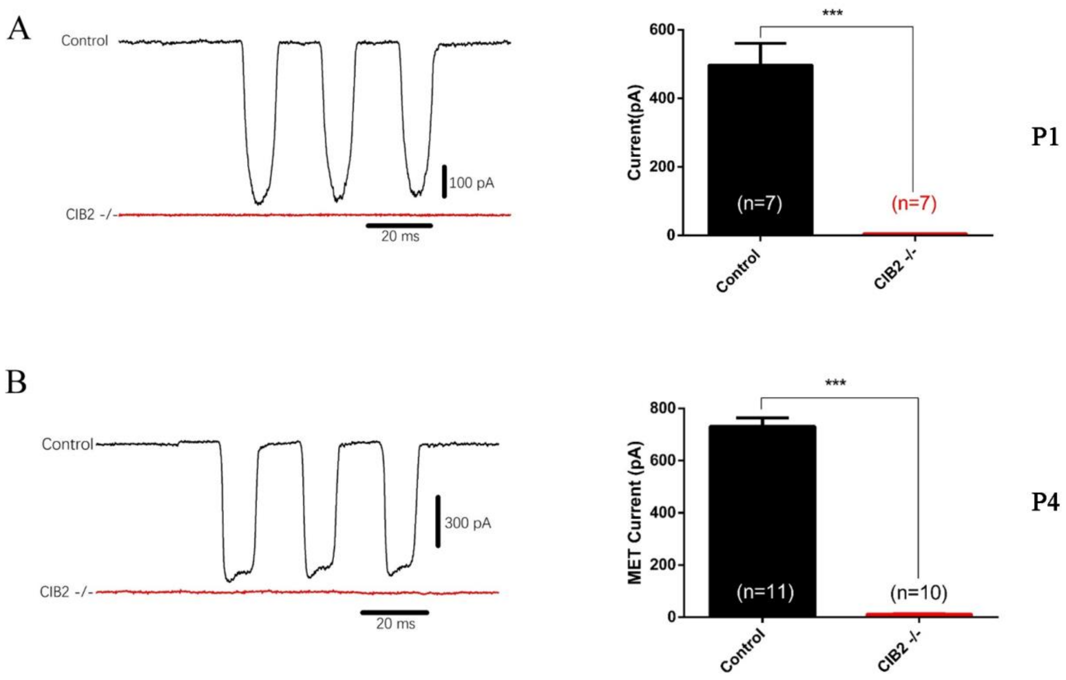

Figure S2. Mechanosensitivity of hair cells is lost in *Cib2* knockout mice. A fluid jet system that drives a sinusoidal deflection of hair bundles was used to evaluate the saturating MET current from hair cells. The average peak current is shown at right. (A) P1 OHCs; (B) P4 OHCs. The differences were evaluated by Student's t-test ($^{***}p < 0.001$).

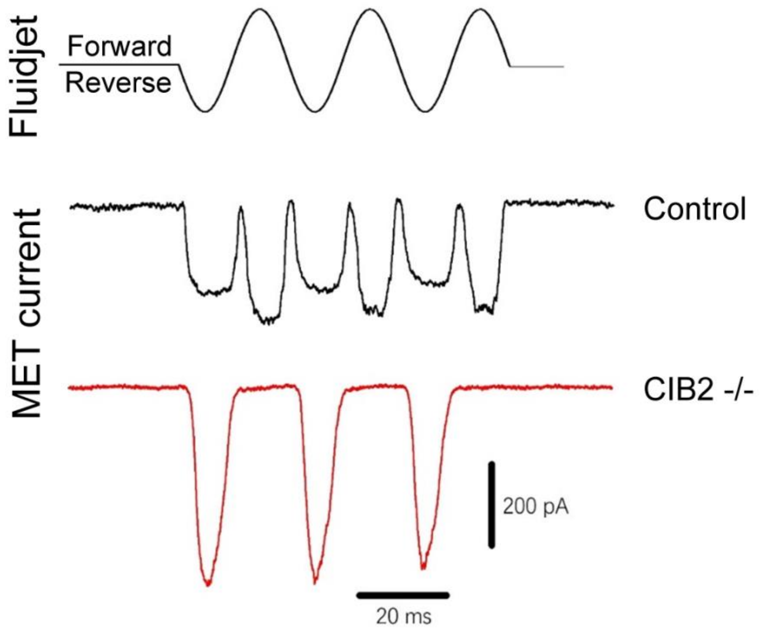

Figure S3. Reverse-polarity mechanosensitivity of hair cells is maintained in *Cib2* knockout mice. A fluid jet system was used to evaluate the conventional as well as reverse-polarity MET current from P4 OHCs.

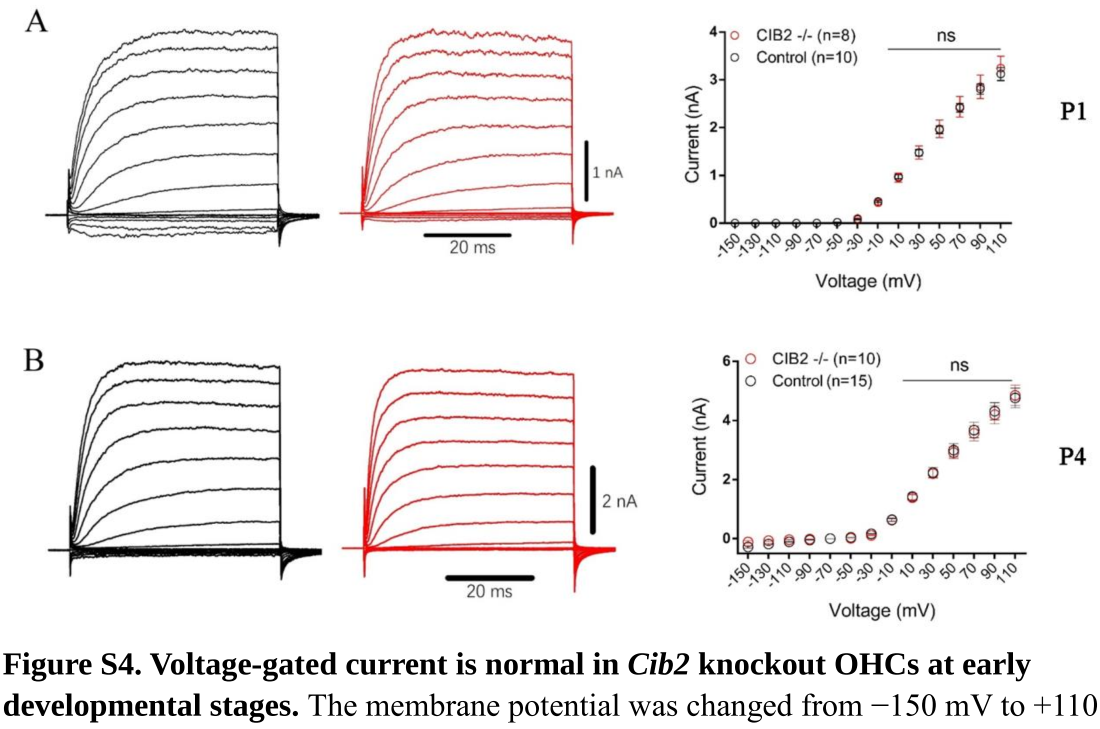

Figure S4. Voltage-gated current is normal in *Cib2* knockout OHCs at early developmental stages. The membrane potential was changed from −150 mV to +110 mV in 20 mV steps, and voltage-gated current was recorded from control and *Cib2* knockout OHCs. Current–voltage (I-V) curves were drawn from data collected from multiple cells. (A) P1 OHCs; (B) P4 OHCs. The differences were evaluated by Student’s t-test (ns, not significant).
